# Geospatial analysis of reported activity locations to identify sites for tuberculosis screening

**DOI:** 10.1038/s41598-022-18456-6

**Published:** 2022-08-18

**Authors:** Courtney M. Yuen, Meredith B. Brooks, Ana Karina Millones, Diana Acosta, Erika Del Águila-Rojas, Hortencia Campos, Sheyla Farroñay, Giannina Morales, Judith Ramirez-Sandoval, Tim C. Nichols, Judith Jimenez, Helen E. Jenkins, Leonid Lecca

**Affiliations:** 1 Division of Global Health Equity, Brigham and Women’s Hospital, Boston, MA USA; 2 Department of Global Health and Social Medicine, Harvard Medical School, Boston, MA USA; 3 Socios En Salud Sucursal Peru, Lima, Peru; 4 Department of Biostatistics, Boston University School of Public Health, Boston, MA USA

**Keywords:** Tuberculosis, Public health

## Abstract

Mobile screening units can help close tuberculosis case detection gaps. Placing screening units where people at high risk for undiagnosed tuberculosis preferentially spend time could make screening more resource-effective. We conducted a case–control study in Lima, Peru to identify locations where people with tuberculosis were more likely to spend time than community controls. We surveyed participants about activity locations over the past 6 months. We used density-based clustering to assess how patient and control activity locations differed, and logistic regression to compare location-based exposures. We included 109 tuberculosis patients and 79 controls. In density-based clustering analysis, the two groups had similar patterns of living locations, but their work locations clustered in distinct areas. Both groups were similarly likely to use public transit, but patients predominantly used buses and were less likely to use rapid transit (adjusted odds ratio [aOR] 0.31, 95% confidence interval [CI] 0.10–0.96) or taxis (aOR 0.42, 95% CI 0.21–0.85). Patients were more likely to have spent time in prison (aOR 11.55, 95% CI 1.48–90.13). Placing mobile screening units at bus terminals serving locations where tuberculosis patients have worked and within and around prisons could help reach people with undiagnosed tuberculosis.

## Introduction

Prior to the COVID-19 pandemic, around 30% of the estimated 10 million people who developed TB annually were not diagnosed, and the pandemic has exacerbated this case detection gap^1^. Community-based active case-finding is an important strategy for closing this gap^2,3^. Mobile TB screening units have long been used for community-based active case-finding^3^. These screening units aim to make TB diagnostic services more accessible and thus increase the numbers of people evaluated for TB.

In settings where TB is endemic, programs face a choice of where to place mobile TB screening units, as TB risk is not uniform within communities. Even within a compact geographic area, yields of TB diagnoses at screening units can vary substantially between adjacent neighborhoods^4^. One common approach to selecting screening unit locations is to map the residences of known TB patients and place screening units in neighborhoods with large numbers of patients^5,6^. Another is to consult community leaders about where they believe people at high risk for TB in their community can best be reached^7,8^. While both these approaches have advantages, they also have limitations. For instance, individuals’ areas of residence may not be the most convenient place for them to access a screening unit if they work during the day when the screening units are likely to be operating. Moreover, community leaders’ beliefs about who is at high risk for TB may not be completely accurate, particularly where TB is a stigmatized condition.

Objective measures of where people with undiagnosed TB are more likely to be found would be valuable to inform the placement of mobile TB screening units. Screening units are more resource-efficient if they preferentially reach people with undiagnosed TB as opposed to low-risk members of the community. Prior studies have assessed where patients spend time in TB-endemic settings, generally in the context of identifying potential sites of transmission that link genetically clustered cases^9–13^. Several of these studies found that genotypically clustered cases shared health care venues^9,10,12^ and occasionally social venues^9,12^. In addition, a study that compared patients with multidrug-resistant TB to a control group without TB found that patients were more likely to report having recently utilized health care venues and public transportation^12^. While these findings are suggestive, these studies were not designed to directly address the question of what types of locations could be prioritized to make screening units more efficient for the general population of people affected by TB. Some focused only on people with multidrug-resistant TB^10,12,13^, and some did not include non-TB control groups^9–11^, meaning that it is not clear whether identified sites are more likely to be frequented by people with TB than people without TB.

To address this knowledge gap, we conducted a case–control study to identify locations where a community-based mobile TB screening unit might be able to reach a population with a disproportionately high risk of undiagnosed TB. We sought to identify places where people with undiagnosed TB were more likely to have spent time than their neighbors who have a low risk of TB.

## Methods

### Study design

We conducted a case–control study comparing locations where TB patients habitually spent time prior to their diagnosis to locations where individuals who live in the same community but who have low risk for TB spent time. Cases comprised adults with pulmonary TB, as these are the individuals most readily diagnosed via mobile TB screening units. The low-risk control group comprised individuals from the same communities without known TB exposure and without TB infection. We aimed to recruit 110 patients and 80 control group participants to give us 80% power at an alpha level of 0.05 to detect a 20% difference in a location-based exposure between groups.

### Setting

Peru is a middle-income country with an estimated TB incidence of 116 per 100,000 population, the highest in Latin America^1^. The metropolitan area of the capital, Lima, has around 10 million residents and is divided into five major regions: Central, North, East, and South Lima, and Callao (Supplementary Fig. S1)^14,15^. Indicators of urbanization and socioeconomic status are higher in Central Lima than the other regions^14,15^. This study focuses on residents of Carabayllo District in the North Lima region. Carabayllo is served by 12 public health facilities operated by the Ministry of Health, which divide the district into defined catchment areas; TB patients receive treatment at the health facility in whose catchment area they reside.

This study was implemented during the COVID-19 pandemic in Peru. Peru’s first case of COVID-19 was diagnosed in March 2020, and a national lockdown was imposed that month^16^. Overall population mobility reduced substantially during the initial lockdown but gradually increased throughout the remainder of the year, although various measures aimed at reducing mobility (e.g. school closures, transportation restrictions) and social contact were still being implemented^17^. The first COVID-19 wave peaked in August 2020, and the second wave peaked in March–April 2021^18^.

### TB patient recruitment

We recruited adult (≥ 18 years old) TB patients with pulmonary TB from 5 centrally located health facilities with contiguous catchment areas during October 2020-June 2021. Together, these catchment areas comprise an area of 17 km^2^, henceforth referred to as the study area. The estimated population of the study area was around 178,000 based on the 2017 census^19^. Patients were recruited within 1 month of treatment initiation. There were no exclusion criteria related to whether the diagnosis was bacteriologically confirmed versus not, patient treatment history, or drug resistance. During the recruitment period, patients were predominantly diagnosed by passive case-finding since all community-based active case-finding activities had been suspended during the pandemic. While active case-finding activities gradually resumed during the final three months of recruitment, we did not distinguish between patients diagnosed through active versus passive case-finding.

### Control group recruitment

Our main consideration in choosing a control group was that they represented low-risk individuals who live in the same communities as the TB patients, as these are the individuals who community-based screening programs want to avoid screening. An ideal control group would have been a random sample of individuals from the same communities as the TB patients who have never had TB and would never develop TB in their lifetimes. Since it is impossible to know who will develop TB in the future, we attempted to select individuals with low TB risk based on their having no prior history of TB or TB contact, as well as their being uninfected with *M. tuberculosis*.

Adults who lived in the study area were eligible for recruitment into the control group. Enrollment took place during December 2020-March 2021. Control group participants were recruited via IRB-approved posters and flyers distributed throughout the study area. As control group enrollment progressed, the study team targeted flyer distribution to address any imbalances in sex and age. People interested in enrollment were screened for a prior history of TB or prior close contact with a friend or family member with TB. Those who reported neither were enrolled, and a tuberculin skin test (TST, the only widely available test for TB infection in Peru) was administered. Participants with a negative TST result (induration < 10 mm) were included in the control group. Participants with a positive TST result were excluded, and study staff coordinated a full TB evaluation.

### Data collection

Study staff familiar with the study area administered a structured survey to participants. The survey asked participants about living, work, educational, and social locations where they spent a substantial amount of time in during the 6 months prior to diagnosis (for patients) or prior to the survey (for control group participants). A recall period of 6 months was used given the months-long diagnostic delays that had previously been reported in Lima^20^, and because responses reflecting this longer period would be less affected by periodic COVID-19-related lockdowns. Living locations included places where participants had officially resided as well as other residences where they had slept overnight or spent most of the day (e.g., staying with family members). Social locations were places apart from homes or work locations where a person spent more than 5 h per week. For each of the locations reported, study staff recorded sufficient information to identify its approximate location on a map; locations were described using combinations of streets and landmarks or using a local street-and-block system, as many places do not have numeric addresses. Thus, mapped locations are likely to be accurate at the level of a city block.

Participants were also asked about usage of public transit, health facilities, and congregate settings, as these are types of locations that could be served by a mobile TB screening unit. We asked about how frequently participants used public transit over the past 6 months and which modalities they commonly used. Modalities included a government-operated rapid transit network, independently operated minibuses and buses that have established routes and stops, and taxis or moto-taxis (auto-rickshaws) that are individually hired for transport to the client’s preferred destination. We asked what health facilities participants normally used, not restricted to the past 6 months. We later classified facilities into the following categories: public Ministry of Health facilities, government health facilities serving people with employer-based health insurance, public–private partnership hospitals operated by the Municipality of Lima, and the private sector. Finally, we asked whether participants had ever spent time in prisons or rehabilitation centers, or lived in any other place with congregate living arrangements such as a dormitory.

We did not collect information on individual characteristics or risk factors, as our objective was to identify locations where it would be useful to place screening units, not to identify individual risk factors for TB. Individual risk factors for TB are well established, including both medical comorbidities (e.g. HIV, diabetes) and social determinants (e.g. income, housing instability). We know that TB patients are likely to differ from their low-risk neighbors with respect to these factors, and some of these factors may correlate with or cause differences in where people spend time. We did not want to control for these risk factors as potential confounders because the programmatically useful outcome for this analysis is the difference in activity locations between the two groups, regardless of the underlying cause.

### Analysis

The study staff who conducted the surveys mapped the approximate locations of the reported living, work, educational, and social locations on Google Maps. Coordinates were exported from Google Maps and overlaid onto a shapefile of the study area using ArcGIS Pro Version 2.8.0 (Environmental Systems Research Institute, Redlands, California, USA). We used the high density-based spatial clustering of applications with noise (HDBSCAN) algorithm, specifying a minimum cluster size of 5, to explore the presence of coherent clusters of locations at which individuals reported. We chose the HDBSCAN method because it allows for the identification of clusters of arbitrary shapes and can identify outliers. It requires a single input parameter of minimum cluster size, which may aid in the identification of clusters missed by other density-based clustering methods that rely on a predefined distance threshold.

We used logistic regression to assess differences between location-based exposures reported by TB patients and control group participants, adjusting for sex. Analysis was performed using SAS v9.4 (SAS Institute, Cary, NC, USA).

### Ethics declaration

This study was conducted in accordance with the U.S. Health and Human Services regulations for the protection of human subjects (HHS 45CFR 46). All participants were enrolled with written informed consent. This study was approved by the Mass General Brigham Institutional Review Board (protocol 2019P003679) and the Ethics Committee of the Universidad Peruana Cayetano Heredia (protocol 19,022).

## Results

During October 2020-June 2021, we enrolled 112 adults with pulmonary TB from the study area’s 5 public health facilities. These patients represented 90% of the adult pulmonary TB patients registered for treatment in these health facilities during this period. Two patients decided not to be surveyed after enrollment, and one patient was excluded from the analysis when the survey revealed that he did not reside in the study area and was registered for treatment using a relative’s address. Thus, 109 (97%) patients remained in the analysis. We concurrently enrolled 86 adults living in the same catchment areas who reported no history of TB disease or contact, of whom 79 (92%) were included in the control group following a negative TST result. There was a greater proportion of males among the included TB patients than the control group, and the age distribution was comparable in both groups (Table 1).

**Table 1:** Characteristics of pulmonary TB patients (N = 109) and TST-negative community control group participants (N = 79).

		TB patients, n (%)	Community controls, n (%)	Chi square *p* value
Sex	Female	42 (39)	38 (48)	0.190
	Male	67 (61)	41 (52)	
Age group	18–39	67 (61)	49 (62)	0.960
	40–59	32 (29)	22 (28)	
	60 +	10 (9)	8 (10)	

### Geospatial analysis

In surveys, the 109 patients reported 159 living locations (range 1–3 per person), 63 work locations (range 0–2 per person), and 40 social locations (range 0–4 per person) where they spent time in the 6 months prior to diagnosis. The 79 control group participants reported 113 living locations (range 1–3 per person), 67 work locations (range 0–3 per person), and 27 social locations (range 0–2 per person) where they spent time in the 6 months prior to the survey. No participants reported attending educational institutions, as these were closed due to the COVID-19 pandemic.

**Figure 1:**
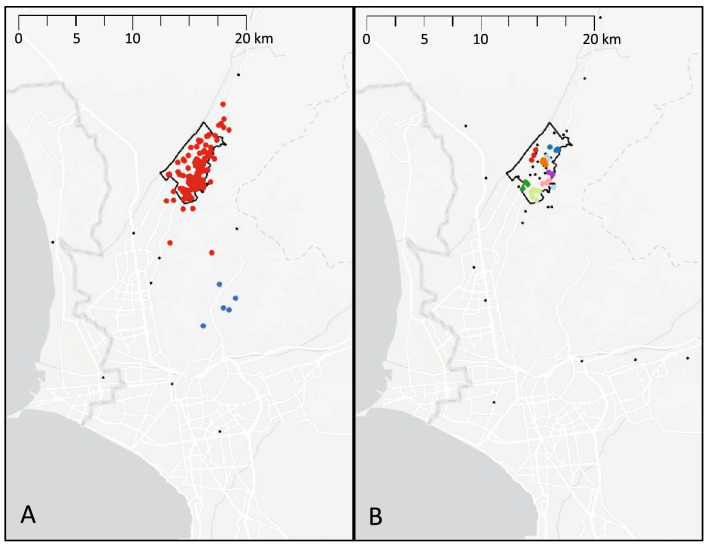
Density-based clustering of locations where participants reported having lived, slept, or spent the majority of the day during the past 6 months. Each dot represents a location reported by a study participant, with colors representing clusters and black dots representing non-clustered locations. The black outline indicates the study area. Reported locations mainly clustered in the study area for both (**A**) TB patients (red dots) and (**B**) control group participants (microclusters shown in different colors). Map was created by MBB using ArcGIS Pro Version 2.8.0 (Environmental Systems Research Institute, Redlands, California, USA).

**Figure 2:**
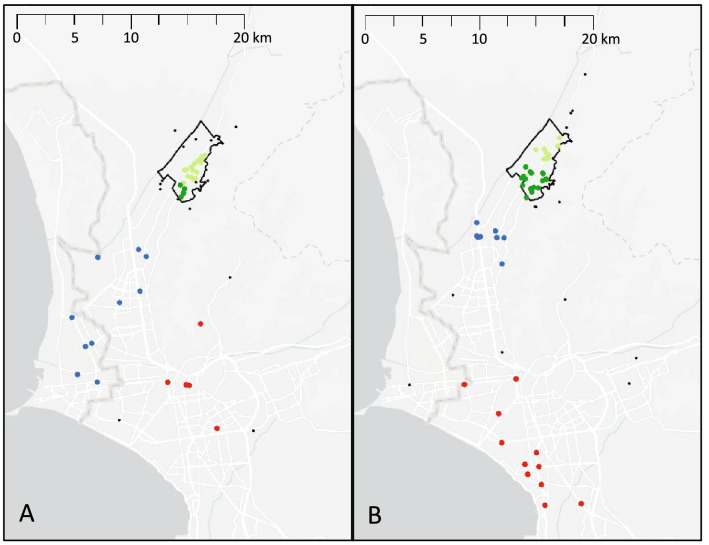
Density-based clustering of locations where participants reported having worked during the past 6 months. Each dot represents a location reported by a study participant, with colors representing clusters and black dots representing non-clustered locations. The black outline indicates the study area. (**A**) For TB patients, locations clustered around the main avenue within the study area (green dots) with another large but disperse cluster closer to the port of Callao (blue dots). (**B**) For control group participants, clusters were identified within the study area (green dots), in a neighboring district (blue dots), and in the more affluent Central Lima region (red dots). Map was created by MBB using ArcGIS Pro Version 2.8.0 (Environmental Systems Research Institute, Redlands, California, USA).

**Figure 3:**
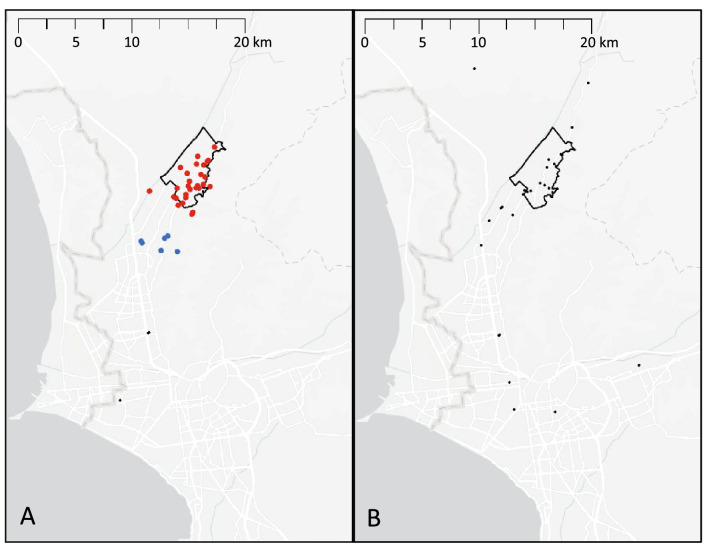
Density-based clustering of locations where participants reported having socialized routinely during the past 6 months. Each dot represents a location reported by a study participant, with colors representing clusters and black dots representing non-clustered locations. The black outline indicates the study area. (**A**) Among TB patients, locations clustered mainly within the study area (red dots) with a smaller cluster in a neighboring district (blue dots). (**B**) No clusters were identified for control group participants. Map was created by MBB using ArcGIS Pro Version 2.8.0 (Environmental Systems Research Institute, Redlands, California, USA).

Patterns in living locations in the past 6 months were similar for patients and control group participants (Fig. 1). The two groups were similar in terms of the average number of living locations (1.46 for patients, 1.43 for control group participants, *p* = 0.759), as well as percentage of participants who reported locations outside the study area (29% for both groups, *p* = 0.969). Visual comparison of density-based clustering suggested that work locations differed between the two groups (Fig. 2). While there were large clusters of work locations in and around the study area for both groups, these clusters centered along the major avenue in the district for patients, while they were more dispersed for control group participants. Work locations outside the study area also clustered in different regions. For example, a cluster of patient work locations was located southwest of Carabayllo toward the port of Callao, while a cluster of control group work locations centered on the more affluent Central Lima region. No clusters of social locations were identified for control group participants (Fig. 3). For patients, a large cluster of locations centered on the study area, while a smaller cluster was located in a neighboring district.

### Public transit, health facilities, and congregate settings

Around half of both the patients (52%) and control group participants (56%) reported regular public transit use, but modes of transit differed (Table 2). Patients predominantly reported using buses, and they were significantly less likely to report using the rapid transit network (aOR 0.31, 95% CI 0.10–0.96) or taxis (aOR 0.42, 95% CI 0.21–0.85) than control group participants. The two groups also differed with respect to what types of health facilities they reported using. Differences in usage of the Ministry of Health network and the employer-based insurance network were an expected result of our sampling strategy, as we only enrolled TB patients from the Ministry of Health network. However, we also found that private sector health facility usage was higher among TB patients (22%) than among control group participants (15%), although this difference did not achieve statistical significance (aOR 1.65, 95% 0.76–3.56). Finally, patients were significantly more likely than control group participants to report having spent time in a prison (14% vs 1%, aOR 11.55, 95% CI 1.48–90.13).

**Table 2:** Usage of public transit, health facilities, and congregate residential locations by pulmonary TB patients (N = 109) and community control group participants (N = 79).

Locations where participants spent time	TB patients, n (%)	Community controls, n (%)	Sex-adjusted odds ratio (95% CI)	95% CI
Any regular public transit use (at least once a week during the past 6 months)	57 (52)	44 (56)	0.86	(0.48–1.55)
Regular public transit use and typically uses the rapid transit network	5 (5)	11 (14)	0.31	(0.10–0.96)
Regular public transit use and typically uses minibuses or buses	49 (45)	31 (39)	1.26	(0.69–2.27)
Regular public transit use and typically uses taxis or moto-taxis	18 (17)	25 (32)	0.42	(0.21–0.85)
Typically uses Ministry of Health facilities	82 (75)	47 (59)	2.13	(1.14–4.01)
Typically uses employer-based insurance network health facilities	1 (1)	13 (16)	0.04	(0.01–0.35)
Typically uses public–private partnership health facilities	12 (11)	8 (10)	1.19	(0.46–3.68)
Typically uses private health facilities	24 (22)	12 (15)	1.65	(0.76–3.56)
Ever spent time in a prison	15 (14)	1 (1)	11.55	(1.48–90.13)
Ever spent time in a rehabilitation center	8 (7)	2 (3)	2.82	(0.58–13.75)
Ever spent time in another congregate residential setting	11 (10)	5 (6)	1.48	(0.48–4.53)

## Discussion

We found that patterns of activity locations were different for TB patients compared to people who live in the same communities but who have a low risk of TB. Compared to a low-risk control group, TB patients worked and socialized in different places, and they used different forms of public transport. TB patients were far more likely to have spent time in a prison. Some of these locations might themselves be associated with TB transmission, while other differences in activity locations might reflect underlying socioeconomic or behavioral differences between high- and low-risk populations without being associated with transmission. Ultimately, locations that are preferentially frequented by people at high risk for TB disease are potentially useful as screening venues regardless of whether they are sites of transmission or not. Our results suggest that decisions on where to place mobile TB screening units could be aided by considering work-related locations and public transit usage, as well as focusing on people who pass through the prison system.

Work-related activities may be particularly useful for informing placement of mobile TB screening units, as work locations differed between TB patients and their low-risk neighbors, and people’s working hours often coincide with the screening units’ hours of operation. Indeed, a TB screening program in North Lima found that mobile TB screening units in residential and community settings disproportionately attracted older adults, while screening units at public transit terminals used by commuters were better attended by working-age adults^21^. Our study did not observe an association between public transit usage and TB, which has been reported in other studies^12,22,23^. However, we found that patients used different types of public transit than control group participants. Given that the geographic clustering of work locations also differed between the two groups, these results suggest a possible strategy of placing screening units at public transit terminals that serve areas where patients are more likely to work.

Our findings also underscore the importance of improving TB care for people who pass through the prison system. TB incidence in prisons has been estimated to range from 4 to 27 times higher than in the general population, varying across regions of the world^24^. Crowding, poor ventilation, and lack of access to health care fuel the spread of TB within prisons^25^. We cannot say for certain that the 14% of TB patients in our study who reported a history of incarceration were infected with TB while in prison. However, previous studies have shown that prisons serve as a reservoir for ongoing introduction of TB into the community^26,27^, and that the neighborhoods directly surrounding prisons have elevated TB incidence^27^. It is thus imperative to improve TB services within prisons, including regular screening (which can be aided by mobile TB screening units^28^), access to treatment for TB disease and infection, and referral mechanisms to ensure continuity of treatment when people with TB enter or exit the prison system. In addition, placing mobile TB screening units in neighborhoods surrounding prisons and where people tend to move after release could help diagnose people whose TB is currently being missed within the prison system.

The fact that over a quarter of patients in our study had lived or spent substantial amounts of time in homes outside the study area highlights the need for cross-jurisdictional contact tracing mechanisms. Contact tracing is often the responsibility of staff at the health facility where patients receive treatment, and these staff may lack a mechanism to evaluate contacts who live in other catchment areas or districts. This means that high-risk close contacts may not be evaluated or given preventive treatment, even if the patient has informed the health system about them. Electronic platforms can help to facilitate cross-jurisdictional contact tracing and close gaps in contact management by allowing staff in one jurisdiction to alert staff in another jurisdiction about the need to evaluate a contact^29^. While we found that patients were similar to control group participants in terms of having lived outside the study area in the past 6 months, other studies have found TB patients to be less mobile on a short-term basis than community controls^13,30^. It is possible that our study’s comparatively long recall period might have revealed instability in long-term housing as opposed to the day-to-day mobility measured by other studies. Further research is needed to better understand housing stability of TB patients in high-burden settings, as housing instability can promote transmission and complicate contact investigations^31,32^.

Our analysis was subject to both strengths and limitations related to the control group. We recruited control group participants through a broad community-based strategy focused on the neighborhoods where patients lived, as we had no sample frame from which to base a random sample. Although our control group was not a random sample, its age and sex distribution was similar to that of the area as a whole^4^. This strategy had the strength of avoiding the certain selection bias that would arise from recruiting only specific types of people into the control group such as community health workers or people in care at public health facilities, who have been used as community controls in other studies of this type^12,30^. However, because we do not know how our control group would have compared to a random sample of TST-negative community controls, we were unable to assess the bias introduced by our sampling strategy.

Other important limitations include the fact that COVID-19-related lockdowns in Peru likely affected patterns of movement during the recall period. However, the lockdowns affected both groups, and despite the lockdowns, many participants in both groups reported work locations, social locations, and public transit use. Thus, the differences that we observed are likely valid, but reduced mobility might have prevented us from identifying some locations where people would have spent time in the absence of the pandemic. Moreover, because active case-finding activities were suspended during most of the enrollment period, we were unable to assess whether activity locations differed between patients diagnosed through passive versus active case-finding. Second, our sample size was also relatively small, as we powered the study to detect large differences that would likely be meaningful in making community-based screening programs more efficient. However, this meant our study was underpowered to assess smaller but interesting differences in behavior, or to assess more granular information about specific public transit lines, health facilities, or other activity locations. Finally, our question about health facility usage referred only to formal health care settings, which are common venues for TB screening activities. While this question captured small private general practitioner officers that are often ignored in public sector-focused research, it failed to capture informal health care settings such as pharmacies and traditional healer services, which could also prove to be useful screening venues.

A strength of our study was the use of density-based clustering of mapped locations as opposed to attempting to identify common named venues. This allowed us to uncover geographic patterns that differentiated TB patients from control participants, and we were able to make full use of participant responses despite participants frequently being unable to recall place names such as private clinics or social venues. Our choice of the HDBSCAN method had the advantage of being able to identify non-spherical clusters of diverse sizes, which may be missed by other methods. While HBDSCAN may identify a larger number of micro-clusters when fewer clusters might be more representative of location, we tested several minimum cluster sizes to ensure that our conclusions were robust to changes in this parameter.

In conclusion, our study demonstrates that even within communities where TB is endemic, people at high risk for TB may spend time in different places than people at low risk for TB. These differences could be useful for informing the placement of mobile TB screening units, with the goal of preferentially reaching people with undiagnosed TB. TB programs could consider regularly collecting or periodically surveying TB patients about where they have recently worked to identify locations that might benefit from mobile TB screening units. Using mobile TB screening units in prisons and the areas that surround them is also likely to be a relatively high-yield activity. However, simultaneous collaboration with the justice system to improve TB treatment and prevention within the prison system is necessary to ensure that these screening programs have an impact on TB incidence. Ultimately, optimizing the use of mobile TB screening units based on local knowledge of TB epidemiology will help to close the case detection gap and ensure that people with TB are promptly diagnosed and treated.

## Supplementary Information

Supplementary Information.

## Data Availability

Datasets analyzed during the current study are not publicly availability to avoid revealing information about participant residences or work locations, but are available from the corresponding author on reasonable request.
