# Supplementary material for: Geospatial analysis of reported activity locations to identify sites for tuberculosis screening
Source: Sci Rep. 2022 Aug 18;12:14094. doi: 10.1038/s41598-022-18456-6 (PMC9387880; doi:10.1038/s41598-022-18456-6)

Figure S1: Regions of Lima and Callao in relation to the study area. The grey area along the left and bottom of map is the Pacific Ocean. Map was created by MBB using ArcGIS Pro Version 2.8.0 (Environmental Systems Research Institute, Redlands, California, USA)

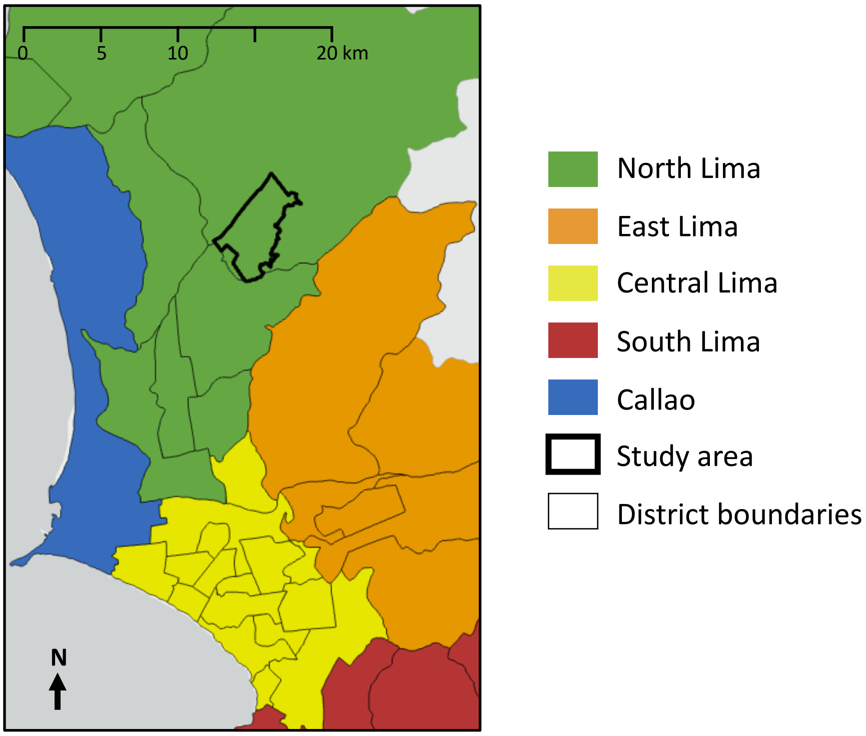

Supplement: Supplementary file 1 — Supplementary Information. [file 41598_2022_18456_MOESM1_ESM.pdf]
